# Prognostic and clinicopathological value of perineural invasion in colorectal cancer: a meta-analysis

**DOI:** 10.3389/fonc.2026.1910736

**Published:** 2026-09-09

**Authors:** Xinyue Zhang, Jingtong Tang, Jianping Zhou

**Affiliations:** Department of Gastrointestinal Surgery, The First Hospital of China Medical University, Shenyang, China

**Keywords:** colorectal cancer, meta-analysis, perineural invasion, prognosis, prognosis factors

## Abstract

**Background:**

Perineural invasion (PNI) is a pathological feature associated with aggressive tumor behavior in several solid malignancies. In colorectal cancer (CRC), PNI has been reported to correlate with poorer prognosis, as well as increased risks of postoperative recurrence and metastasis. However, the available evidence regarding the prognostic significance of PNI in CRC remains limited and inconsistent. Unlike previous meta-analyses that included studies across broader time span. This study focuses on recent evidence and specifically investigates the sources of heterogeneity and its clinical significance. Therefore, this study aimed to evaluate the prognostic and clinicopathological value of PNI in CRC.

**Methods:**

The PubMed, Web of Science, Embase, and Cochrane Library databases were all thoroughly searched between January 1, 2020, and October 9, 2025.Studies included in this review comprised cohort, case–control that reported on PNI status and survival outcomes in CRC. Two reviewers independently extracted the data and evaluated the studies’ quality in accordance with PRISMA recommendations. The Newcastle–Ottawa Scale was used to evaluate the studies’ quality. By computing the hazard ratio (HR) and its 95% confidence interval (CI), the prognostic significance of PNI for CRC was evaluated.

**Results:**

A total of 9 retrospective studies were included. The pooled analysis suggested that PNI was associated with worse OS (HR = 2.23, 95%CI: 1.48-3.35, P < 0.001) and DFS (HR = 1.94, 95%CI: 1.44-2.64, P < 0.05) in patients with CRC. However, substantial heterogeneity was observed in both analyses (OS: I²=79.7%; DFS: I²=67.4%), and publication bias was suggested by Egger’s tests (OS: p=0.0097; DFS: p=0.0085).

**Conclusions:**

Based on recent studies, PNI was associated with poorer OS and DFS in CRC and may serve as a useful pathological marker for risk stratification. However, the findings should be interpreted cautiously because all included studies were retrospective and substantial heterogeneity and potential publication bias were present.

**Systematic review registration:**

https://www.crd.york.ac.uk/PROSPERO/, identifier CRD420251161522.

## Introduction

In terms of incidence and mortality, colorectal cancer (CRC) is one of the most common malignant tumors in the world (1, 2). CRC is becoming more frequent; by 2040, there will be 3.2 million cases globally. creating an increasing global health burden to public health (3). For patients with metastatic colorectal cancer (mCRC), attaining long-term survival and efficient disease management continues to be a major therapeutic challenge, even with advancements in multimodal treatment approaches and customized chemoradiotherapy regimens (4). Immunotherapy has become a promising treatment option for CRC in recent years, although patients with advanced and metastatic CRC still have a dismal prognosis (5). The Tumor-Node-Metastasis (TNM) staging method is currently considered the gold standard for prognostic evaluation and therapy recommendations (6). However, clinically significant variations in prognosis are often observed among patients with identical TNM staging, indicating that the current assessment system remains imperfect. The search for new prognostic indicators to complement TNM staging is still continuing (7, 8). A large-sample study based on the NCDB data shows that the current AJCC 8th edition TNM staging for colorectal cancer is not completely hierarchical in terms of survival stratification. For some patients, even though their staging is different, their mortality risks still overlap. This suggests that relying solely on the traditional TNM staging may not accurately reflect the true prognosis (9). Therefore, it is necessary to combine supplementary indicators such as PNI, which can reflect the tumor’s invasiveness and biological behavior, to further optimize the risk stratification.

Perineural invasion(PNI) refers to the process where tumor cells invade nerves. This is considered an important route of metastasis in various malignant tumors, including colorectal cancer (10). But, the definition and pathological assessment of PNI are not entirely uniform across studies (11). Sometimes, PNI is defined as the infiltration of tumor cells into the fibrous sheath or the encasement of nerve fibers by cancer cells to a degree that exceeds 33% of their circumference (12). Perineural invasion, according to Fujita et al., is the existence of cancer cells in the perineurium of nerve fascicles in Auerbach’s plexus (13). In addition, the definition of neural infiltration is often prone to confusion. The terms used to describe nerve involvement are not entirely consistent. In particular, intraneural invasion, perineural invasion, and perineural infiltration are related but conceptually distinct phenomena, and their definitions may vary depending on the pathological and clinical context (14). PNI is considered as the third major route of tumor metastasis, distinct from lymphatic and hematogenous spread (15). PNI is an independent negative prognostic factor for several malignant tumors, including prostate, gastric, and pancreatic cancers, and usually signifies that the tumor has a more aggressive invasive biological nature (16–18). In colorectal cancer, the detection rate of PNI varies considerably (ranging from 10% to 33%) largely due to differences in detection methods and pathological assessment criteria (19). Despite the fact that a number of retrospective studies have looked at the connection between PNI and both OS and DFS in patients with CRC, individual studies are frequently hampered by small sample sizes, which results in conclusions that are noticeably inconsistent (20).

Several previous meta-analyses have evaluated the prognostic value of PNI in colorectal cancer. A meta-analysis conducted by Yang et al., which included 38 studies involving a total of 12,661 patients, showed that PNI was significantly associated with poorer overall survival (HR = 2.07, 95% CI: 1.87–2.29) and disease-free survival (HR = 2.23, 95% CI: 1.79–2.78) (21). Another systematic review and meta-analysis focusing on patients with lymph node-negative colorectal cancer found that adjuvant chemotherapy could improve OS (HR = 0.52, 95% CI: 0.40 - 0.69) and DFS (HR = 0.53, 95% CI: 0.35 - 0.82) in patients with positive PNI, but had no significant effect on DFS in patients with negative PNI (22). These previous meta-analysis included studies with a wide time span and given the ongoing evolution of pathological assessment and treatment strategies in colorectal cancer, an updated evaluation based on more recent studies may provide evidence that is more closely aligned with current clinical practice. PNI has been listed by the NCCN guidelines as one of the high-risk features for stage II colorectal cancer, which may influence the decision on adjuvant chemotherapy (23). However, its prognostic value in different stages still needs to be systematically evaluated. Therefore, this meta-analysis aimed to systematically assess the prognostic and clinicopathological value of PNI in CRC, with particular focus on overall survival and disease-free survival, in order to inform risk stratification and clinical decision-making.

## Materials and methods

With registration number CRD420251161522, the study protocol is pre-registered with the International Prospective Systematic Review Registry (PROSPERO). The Preferred Reporting Items for Systematic Reviews and Meta-Analyses (PRISMA) criteria are closely followed in the conduct and reporting of this study.

### Literature search

Four databases were searched: Web of Science, Embase, Cochrane Library, and PubMed. The search was restricted to studies published between January 1, 2020 and October 9, 2025.In order to evaluate the prognostic value of perineural invasion (PNI) in the context of current pathological assessment guidelines and clinical care of colorectal cancer, we have purposefully focused on the research published in the last five years. At the same time, we want to reduce the historical variability resulting from adjustments to staging systems, pathology reporting techniques, and treatment strategies. In addition, the involvement of the tumor neural microenvironment in the development of tumors has progressively become a focus of research in recent years. As a result, the evidence pertaining to PNI during the previous five years is more practically relevant.

The construction of search terms is based on the PICOS principle, and the search terms include “perineural invasion”, “prognosis” and “colorectal cancer”, etc. (For detailed search strategy, see **Supplementary Table 1**).

### Criteria for inclusion and removal

The following are the study’s inclusion criteria: (1) Individuals having pathologically proven colorectal cancer; (2) studies in which PNI status was clearly assessed and reported; (3) observational clinical studies; (4) studies reporting HRs with 95% CIs, or providing sufficient data for effect size extraction.

The exclusion criteria were as follows: (1) reviews, systematic reviews, meta-analyses, conference abstracts, editorials, case reports, or duplicate publications; (2) animal or basic research studies; and (3) studies lacking sufficient outcome data.

### Data extraction and quality assessment

Pre-made inclusion and exclusion criteria were used to screen the literature. Two researchers independently examined the literature and extracted data. The third researcher was consulted in order to settle any disagreements. The literature was managed using EndNote 21 software, which removed duplicates. Additional reading and screening were done for papers whose exclusion could not be identified from the title and abstract. The first author, publication year, country, sample size, gender, age, study length, research methodology, TNM stage, treatment, survival consequence, follow-up time, type of survival analysis, hazard ratios (HRs), and 95% confidence intervals (CIs) are all included in the extraction of literature data. DFS is the secondary objective, while OS is the primary result. Because multivariate estimates better account for possible confounding, they were preferentially retrieved when both univariate and multivariate HRs were available. Unless there was enough information for estimate, studies that merely reported Kaplan-Meier curves without extractable HRs were disregarded.

The subgroup analysis was pre-defined and sought to identify the probable sources of heterogeneity. The analysis was stratified according to the following factors: geographical region (Asia vs. Europe), sample size (≤ 500 vs. > 500), TNM stage (I, II, III, IV, and mixed stage), site of occurrence (colorectal cancer, rectal cancer, and colon cancer), histological type (adenocarcinoma, signet ring cell carcinoma), and treatment modality (surgery alone, surgery combined with radiotherapy and chemotherapy/neoadjuvant radiotherapy and chemotherapy, surgery combined with neoadjuvant radiotherapy and chemotherapy plus liver resection). The subgroup categorization was selected before the study based on clinical importance and existing research.

The Newcastle–Ottawa Scale (NOS) was used in this analysis to evaluate the caliber of the observational studies that were included. The scale has a total score range of 0–9 points and consists of three assessment dimensions: research population selection (0–4 points), group comparability (0–2 points), and outcome measurement (0–3 points). High-quality studies were those that received a total score of at least six.

### Statistical analysis

By combining the included studies, we computed the HRs and its 95% CIs to evaluate the prognostic significance of PNI in CRC. Heterogeneity between studies was evaluated using Cochran’s Q test and the I2 statistic. If there was considerable heterogeneity (I2 > 50% or Q test p-value < 0.10), a random-effects model was used; if not, a fixed-effects model was used. DFS was a secondary endpoint, while OS was the main predictor. Sensitivity analyses were performed to verify the robustness of the results, and Begg’s correlation test and Egger’s linear regression approach were used to investigate publication bias. The trim-and-fill method was used as an exploratory investigation when publication bias was discovered. As advised by the Cochrane Handbook (which typically calls for at least 10 trials per covariate), meta-regression was not carried out since there were not enough included studies (n=9) to produce trustworthy estimates. Rather, to investigate possible sources of heterogeneity, subgroup analysis and leave-one-out sensitivity analyses were carried performed.

R software (version 4.5.2) and its Meta and Metafor packages for meta-analysis were used for all statistical analyses in this study.

## Results

### Study retrieval procedure

**Figure 1:**
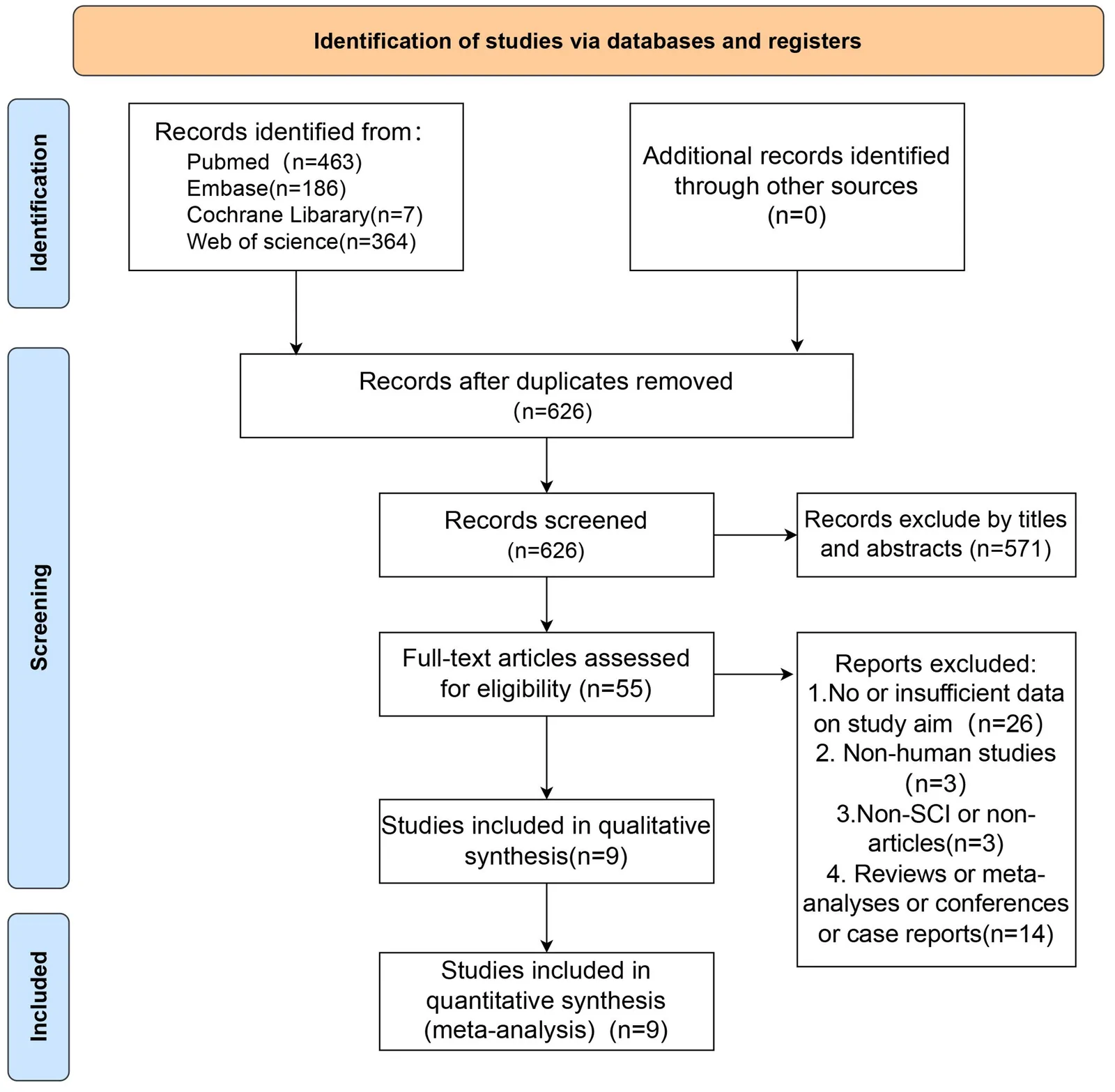
PRISMA flow diagram for the study’s literature search and selection.

A preliminary search yielded 1,020 articles. After removing duplicate entries, 626 articles remained (**Figure 1**). Following an examination of the titles and abstracts, 571 items that were thought to be unrelated to the study’s objectives were eliminated. Among the 55 retained literatures, after reading the full text, 26 literatures were excluded due to incomplete outcome indicators, 3 literatures involving animal studies, 3 literatures not being SCI-indexed or for not being original articles and 14 literatures being systematic reviews, meta-analyses or case reports. Ultimately, 9 literatures were included and NOS scores were conducted (**Figure 1**).

### Features of the included research

The fundamental features of the listed research are compiled in **Table 1** (2, 24–31),nine retrospective English-language studies with sample sizes ranging from 121 to 3,485 cases (median: 720 cases) that were published between 2020 and 2025 were considered in this analysis. The study populations encompassed patients with various types of CRC, with two studies specifically focusing on rectal cancer (27, 31).

**Table 1:** Fundamental features of the included research.

Study	Year	Country	Sample size	Gender (M/F)	Age (year) median (range)	Study period	Study design	TNM stage	Treatment	Survival endpoints	Survival analysis	Follow-uptime(month) Median(range)	NOS score
Liang Chu	2025	China	121	44/77	PNI(-):59 (48.5–67.5)y PNI(+):59 (42.5–68.0)y	2011-2018	retrospective	III-IV	surgery+CRT/nCRT+surgery	OS	Multivariate	NA	6
Yinghao Cao	2020	China	1412	842/570	PNI(-):58.17 ± 11.89y PNI(+):57.31 ± 12.069y	2013-2016	retrospective	I-IV	surgery	OS	Multivariate	first 2 years:3m; next3-5years:6m	7
Milica Stojkovic Lalosevic	2020	Serbia	245	150/95	62.27 ± 10.74y	2014-2016	retrospective	I-III	surgery+CRT/nCRT+surgery	OS,DFS	Multivariate	first 2 years:3m; next3-5years:6m	8
Soo Young Lee	2021	Korea	899	558/331	<70y:523 ≥70y:366	2007-2015	retrospective	II	surgery+CRT/nCRT+surgery	DFS	Multivariate	55 m(1–106 m)	8
Le Qin	2023	China	548	341/207	≤ 60y:310 >60y:238	2014-2018	retrospective	I-IV	surgery+CRT	OS,DFS	Multivariate	40.4 (0.167–74.167) m	6
Seijong Kim	2020	Korea	720	300/420	60(27-90)y	2008-2013	retrospective	I	surgery	DFS	Multivariate	103.5 m(59–139m)	7
Gabriel Zozaya	2023	Spain	344	64/34	60.1 ± 10.7y	2022-2023	retrospective	IV	surgery+nCRT + liver resection	OS,DFS	Multivariate	39.8 m	6
Bin Zhang	2023	China	3485	2123/1362	<50y:852 ≥50y:2633	2013-2016	retrospective	I-IV	surgery+CRT	OS,DFS	Multivariate	61.9 m	6
Young Il Kim	2021	Korea	1156	778/408	PNI(-):56.6 ± 10.3y PNI(+):55.5 ± 10.5y	2000-2010	retrospective	ypStage I-IV	nCRT+surgery	DFS	Multivariate	63 m(55-80m)	8

PNI, perineural invasion; M, male; F, female; OS, overall survival; DFS, Disease-free survival; TNM, tumor-node-metastasis; NOS, Newcastle-Ottawa Scale; CRT, chemoradiotherapy; nCRT, neoadjuvant chemoradiotherapy; ypStage, Post-neoadjuvant therapy pathological Stage.

Two focused on colorectal cancer (28, 29), one study targeting patients with liver metastases from colorectal cancer (24).Regarding disease stage, four studies included patients across all stages from I to IV (25–27, 30). The remaining studies focused on stages III-IV (2)、stagerI-III cases (31)、stages I CRC (29)、stage II (28) or stage IV patients (24). The treatment methods include different combinations such as neoadjuvant therapy, surgery and adjuvant therapy. Six studies found a correlation between OS and PNI in terms of outcome indicators (2, 24–26, 30, 31), The relationship between PNI and DFS was documented in seven studies (24–29, 31). Every study used multivariate regression analysis to determine the HR and its 95% CI. All included studies were of good quality, as indicated by the NOS score, which varied from 6 to 8 (**Supplementary Table 2**).

### PNI and OS

**Figure 2:**
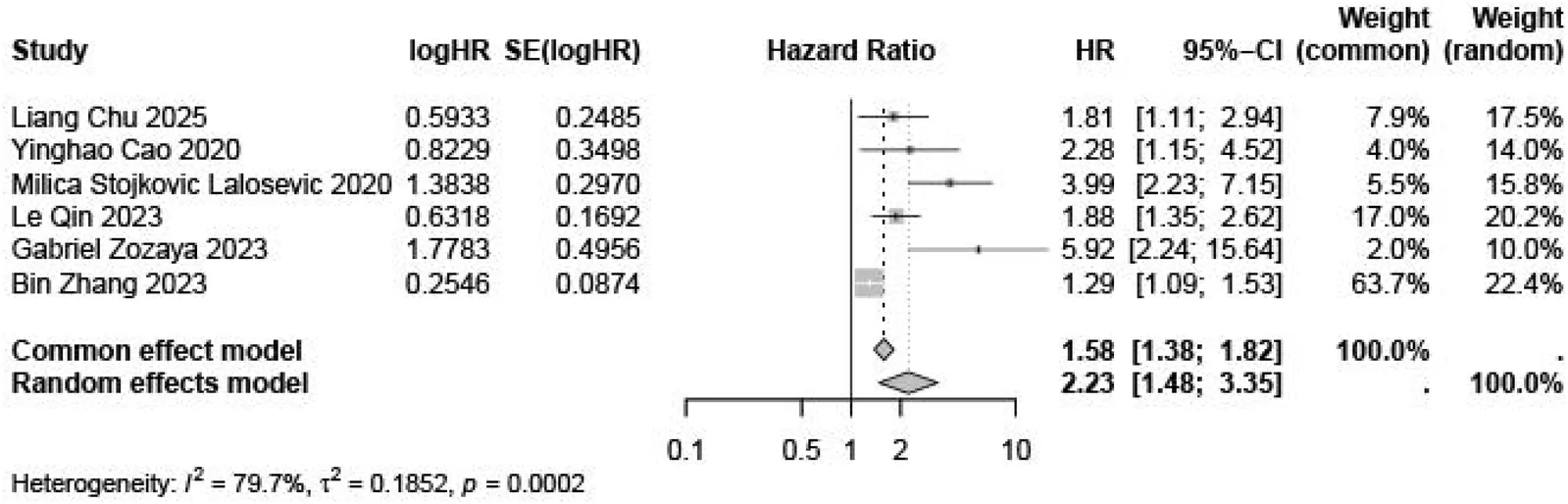
Forest plot of the association between PNI and OS.

Six of the nine included studies (2, 24–26, 30, 31), with 6,155 patients assessed the relationship between PNI and OS in patients with CRC. We selected the random effects model because of the considerable heterogeneity (I2 = 79.7%, p = 0.0002).With a pooled HR of 2.23 (95% CI: 1.48–3.35, P < 0.001), the results show that PNI appears to be a potentially important prognostic marker in colorectal cancer; however, due to the significant heterogeneity and publication bias, its independent prognostic value for overall survival (OS) should be interpreted with caution, indicating a considerably higher risk of death in individuals with PNI (**Figure 2**). PNI is linked to a poor prognosis across TNM staging, histology, and geographic location, according to the subgroup analysis results (**Table 2**). In particular, the European studies showed no substantial heterogeneity (I² = 0%), but the Asian studies showed heterogeneity (I² = 54.6%). Geographical region may be a potential source of heterogeneity, as this difference may be linked to differences in patient profiles, treatment approaches, and healthcare systems across different locations.

**Table 2:** Subgroup analysis of the prognostic value of PNI for OS.

Subgroups	No. of studies	No. of patients	Effects model	HR (95% CI)	P	Heterogeneity	
						I² (%)	Ph
Total	6	6155	Random	2.23 (1.48-3.35)	<0.001	79.7	0.0002
Sample size							
≤500	3	721	Random	3.22 (1.63-6.39)	0.004	70.5	0.0336
>500	3	5434	Random	1.62 (1.16-2.26)	0.002	65.4	0.0554
Geographical region							
Asia	4	5566	Random	1.63 (1.25-2.13)	<0.001	54.6	0.0853
Europe	3	589	Fixed	4.43 (2.69-7.30)	<0.001	0	0.4947
TNM stage							
I-IV	3	5445	Random	1.62 (1.16-2.26)	<0.001	65.4	0.0554
III-IV	1	121	–	1.81 (1.11–2.94)	0.02	–	–
I-III	1	245	–	3.99 ( 2.231-7.148)	<0.05	–	–
IV	1	344	–	5.920 (2.241–15.639)	<0.001	–	–
Histology							
CRC	5	5910	Random	1.92 (1.33-2.77)	0.003	72.3	0.006
RC	1	245	–	3.99 ( 2.231-7.148)	<0.05	–	–
Classification							
Adenocarcinoma	5	6034	Random	2.39 (1.44-3.96)	0.002	83.6	<0.001
SRCC	1	121	–	1.81 (1.11–2.94)	0.02	–	–
Treatment							
surgery	1	1412	–	2.277 (1.147,4.520)	0.0187	–	–
surgery+CRT/nCRT+surgery	4	4399	Random	1.94 (1.25-3.00)	0.003	81.4	0.0011
Surgery+nCRT+Liver resection	1	344	–	5.920 (2.241–15.639)	<0.001	–	–

SRCC, signet-ring cell carcinoma; PNI, perineural invasion; CRC, colorectal cancer; RC, rectal cancer; OS, overall survival; CRT, chemoradiotherapy; nCRT, neoadjuvant chemoradiotherapy;TNM, tumor-node-metastasis.

Regardless of sample size, region, TNM stage, histological type, pathological classification, or treatment modality, subgroup analysis showed that PNI was significantly linked to a lower overall survival. This suggests that PNI is a stable and trustworthy predictor of a poor prognosis in patients with colorectal cancer.

### PNI and DFS

**Figure 3:**
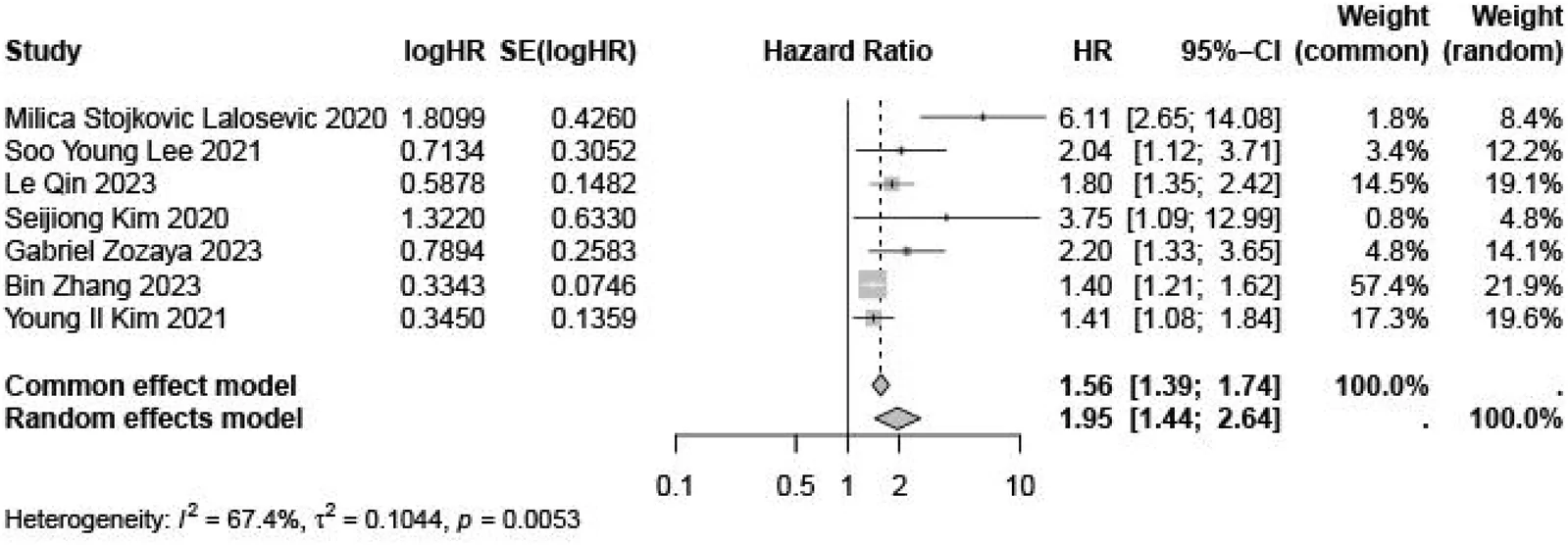
Forest plot of the association between PNI and DFS.

The relationship between PNI and DFS in patients with CRC was assessed in seven of the nine included studies (24–29, 31), encompassing 7,397 individuals (**Figure 3**). We selected the random effects model because of the considerable heterogeneity (I² = 67.4%, p = 0.0053).

To investigate the causes of heterogeneity, we employed subgroup analysis (**Table 3**). First, with HR of 3.47 (95% CI: 1.28–9.37) and 1.49 (95% CI: 1.33–1.67), respectively, small-sample studies (≤500 cases) and large-sample studies (>500 instances) showed a significant connection between PNI and poor prognosis. The large-sample studies showed a notable reduction in heterogeneity (I² = 30.1%), implying that sample size could be a potential source of variability. PNI was substantially linked to worse overall survival in both Asian (HR = 1.49, 95% CI: 1.33–1.67) and European (HR = 3.47, 95% CI: 1.28–9.37) groups, demonstrating that PNI has a consistent prognostic value across several regional populations. PNI was substantially linked to a worse prognosis in all stages, including Stage I (HR = 3.75, 95% CI: 1.09–12.96), Stage II (HR = 2.04, 95% CI: 1.12–3.71), Stages I–III (HR = 6.11, 95% CI: 2.65–14.08), Stages I–IV (HR = 1.46, 95% CI: 1.30–1.64), and Stage IV patients (HR = 2.20, 95% CI: 1.33–3.65). TNM staging may be one of the factors impacting study heterogeneity, as seen by the low heterogeneity (I² = 16.8%) among studies of stages I–IV.

**Table 3:** Subgroup analysis of the prognostic value of PNI for DFS.

Subgroups	No. of studies	No. of patients	Effects model	HR (95% CI)	P	Heterogeneity	
						I² (%)	Ph
Total	7	7397	Random	1.96 (1.44-2.44)	<0.05	67.4	0.0053
Sample size							
≤500	2	589	Random	3.47 (1.28-9.37)	0.007	76.2	0.0405
>500	5	6808	Fixed	1.49 (1.33-1.67)	<0.001	30.1	0.2212
Geographical region							
Asia	5	6808	Fixed	1.49 (1.33-1.67)	<0.001	54.6	0.0853
Europe	2	589	Random	3.47 (1.28-9.37	<0.001	76.2	0.0405
TNM stage							
I	1	720	–	3.751 (1.086–12.957)	0.036	–	–
II	1	899	–	2.041 (1.122–3.712)	0.013	–	–
I-III	1	245	–	6.11 ( 2.651-14.079)	<0.05	–	–
I-IV	3	5189	Fixed	1.46 (1.30-1.64)	<0.001	16.8	0.3005
IV	1	344	–	2.202 (1.327–3.653)	<0.002	–	–
Histology							
CRC	3	4377	Random	1.64 (1.28-2.11)	<0.001	56.7	0.0991
RC	2	1401	Random	2.78 (0.66-11.63)	0.011	90.7	0.0011
CC	2	1619	Fixed	2.29 (1.34-3.92)	0.009	0	0.3865
Treatment							
surgery	1	720	–	3.751 (1.086–12.957)	0.036	–	–
surgery+CRT/nCRT+surgery	5	6333	Random	1.94 (1.25-3.00)	0.003	72.4	0.0059
Surgery+nCRT+Liver resection	1	344	–	2.202 (1.327–3.653)	<0.002	–	–

PNI, perineural invasion; CRC, colorectal cancer; RC, rectal cancer; OS, overall survival; CRT, chemoradiotherapy; nCRT, neoadjuvant chemoradiotherapy; TNM, tumor-node-metastasis.

### Publication bias

**Figure 4:**
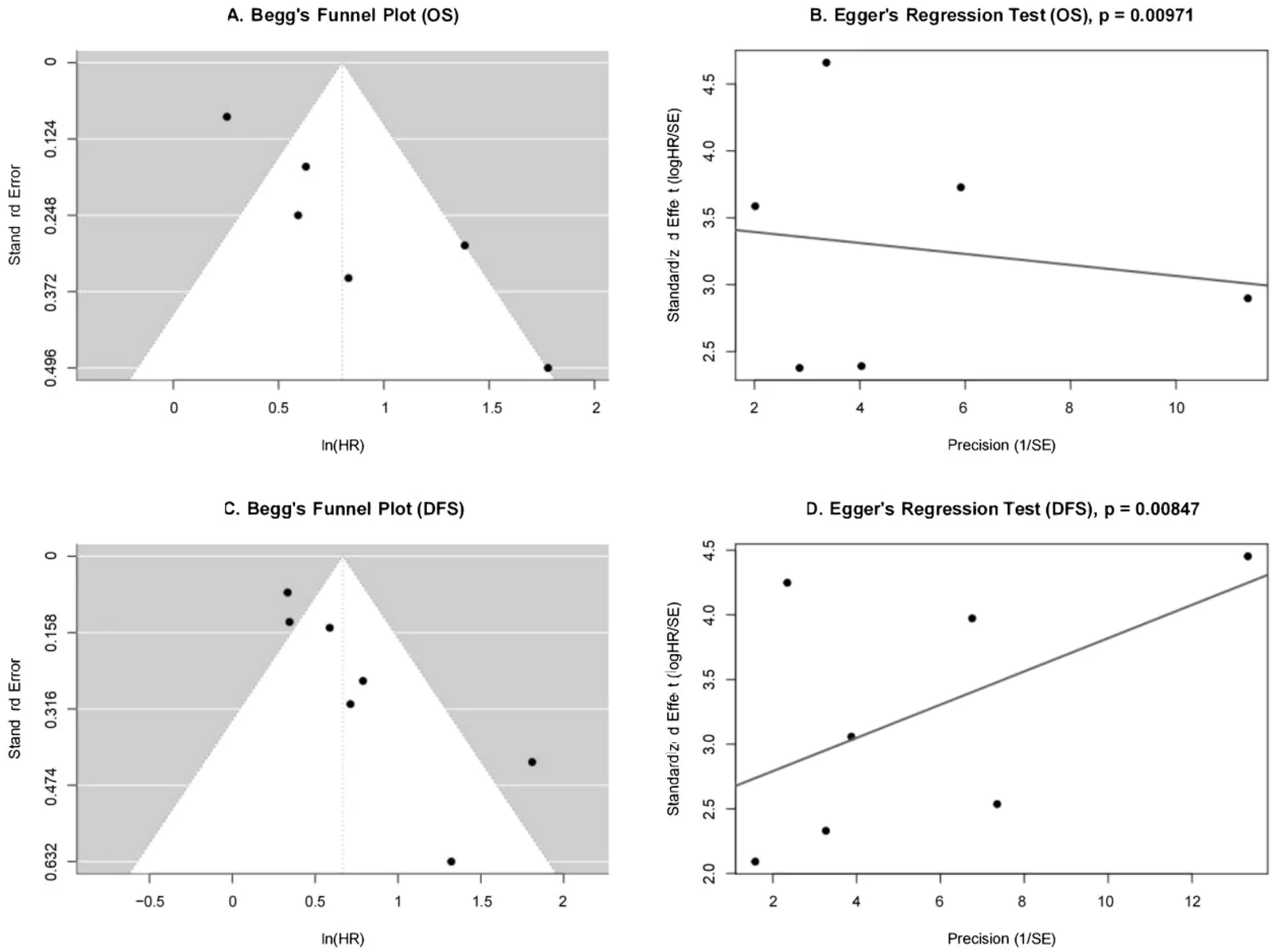
Assessment of potential publication bias using Begg’s funnel plots and Egger’s regression tests. **(A)** Begg’s funnel plot for the OS analysis; **(B)** Egger’s regression test for OS; **(C)** Begg’s funnel plot for the DFS analysis; and **(D)** Egger’s regression test for DFS.

In order to thoroughly evaluate if there was a possible publishing propensity or small sample impact in the research from both the viewpoints of graphic symmetry and statistical testing, this study used funnel plots and Begg’s and Egger’s techniques to detect publication bias. As shown in **Figure 4**, the research points were not evenly distributed in the central area of the funnel plot, but showed a certain degree of offset and asymmetry. Based on the findings identified by Egger’s approach (**Figures 4A, C**), the study’s OS and DFS p values were less than 0.05, at p = 0.00971 and p = 0.00847, respectively (**Figures 4B, D**).The degree of prejudice was rather evident, and the potential of publishing bias was statistically validated. In conclusion, the analysis of publication bias in this study shows that the included studies may have varying degrees of publication bias. Therefore, we adopted the Trim-and-Fill method for analysis to evaluate and adjust the impact of bias on the overall conclusion.

Three missing studies for OS and three for DFS were imputed using the trim-and-fill approach (**Supplementary Figures 1**, **2**). The connection was no longer statistically significant after taking publication bias into account, as seen by the pooled HR for OS becoming 1.50 (95% CI: 0.87–2.59) with the confidence interval crossing unity. The adjusted pooled HR for DFS remained marginally statistically significant at 1.55 (95% CI: 1.01–2.37). These findings suggest that publication bias may have inflated the original effect estimates, and the OS results in particular should be interpreted with caution.

### Sensitivity analysis

**Figure 5:**
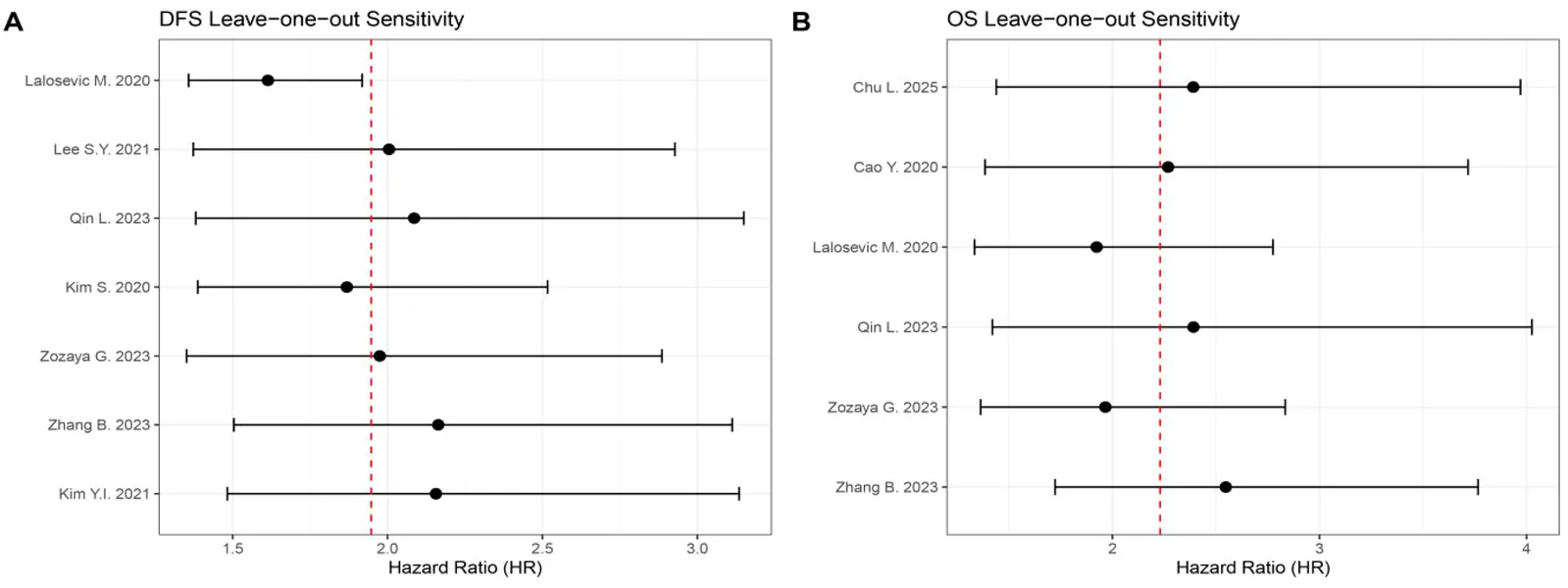
Leave-one-out sensitivity analysis of the meta-analysis evaluating the association between PNI and survival outcomes in colorectal cancer. **(A)** Leave-one-out sensitivity analysis for DFS; **(B)** leave-one-out sensitivity analysis for OS.

To assess the stability of the overall effect size and the influence of different studies on the final outcome, this study employed a leave-one-out sensitivity analysis. In this analysis, each included study was excluded sequentially, and the combined HR was recalculated accordingly. This approach aimed to identify “key studies” or potential outliers that significantly impact the overall results. The findings from the sensitivity analysis (**Figure 5**) indicate that, whether considering DFS or OS, the overall combined effect is highly stable and reliable, unaffected by anomalies in individual studies.

The pooled HR for the OS analysis (**Figure 5B**) varied from 1.86 (95% CI: 1.32–2.62) after omitting Chu L. 2025 to 2.40 (95% CI: 1.58–3.63) after excluding Zozaya G. 2023. The pooled HR declined substantially (from 2.23 to 1.86) when Chu L. 2025 was excluded, indicating that this study might have contributed to the greater effect estimate found in the overall analysis. Nonetheless, the correlation between PNI and OS continued to be statistically significant in every iteration, indicating that no single study was the primary driver of the pooled conclusion. After excluding Lalosevic M. 2020 and Zozaya G. 2023, the pooled HR for the DFS analysis (**Figure 5A**) ranged from 1.74 (95% CI: 1.30–2.33) to 2.01 (95% CI: 1.47–2.74). When we eliminated Lalosevic M. 2020, the study with the highest individual HR (6.11), there was the most change. The total estimate remained significant throughout all rounds despite this variation, indicating that the DFS finding is quite robust and not unduly reliant on any one study.

Although the previous publication bias analysis suggests a degree of publication tendency, the sensitivity results affirm that the core conclusions of this study remain robust and trustworthy.

## Discussion

This study demonstrates a substantial correlation between PNI and poor prognosis based on a meta-analysis of 8,930 colorectal cancer patients. According to the results, people who test positive for PNI have a risk of mortality and recurrence that is more than doubled. The overall survival hazard ratio is (HR = 2.23, 95% CI: 1.48-3.35, P < 0.001), while the disease-free survival hazard ratio is (HR = 1.94, 95% CI: 1.44-2.64, P < 0.05). Furthermore, a review of relevant literature supports that this statistical significance is not coincidental; rather, it is underpinned by complex tumor biological mechanisms (19, 20). Traditionally, nerves have been viewed as low-resistance physical pathways facilitating tumor spread. In contrast, modern oncology increasingly conceptualizes them as bidirectional signaling networks that connect nerves and tumor cells (32). Nerve fibers within the tumor microenvironment can contribute to tumor progression by releasing neurotransmitters and neurotrophic factors (33). For example, it has been shown that glial cell-derived neurotrophic factor (GDNF) plays a significant part in the migration of colorectal cancer cells. By triggering certain signaling pathways, it increases the production of vascular endothelial growth factor, which makes tumors more invasive and anti-apoptotic (34). At the same time, the L1 cell adhesion molecules secreted by tumor cells can also induce nerve axons to grow towards the tumor, forming a vicious cycle (35). The remaining microscopic nerve fibers may continue to shield the dispersed tumor cells from immune surveillance and the effects of chemotherapy (32, 36). This molecular-level interaction may help explain why PNI-positive tumors still have an extremely high recurrence rate after resection.

In addition to its statistical prognostic value, PNI also holds practical significance in clinical diagnosis and treatment for colorectal cancer. One study has suggested that the occurrence of PNI may be associated with molecular characteristics such as KRAS mutations, BRAF mutations, and MSI status, indicating a potential link between PNI and the molecular heterogeneity of colorectal cancer (37). Therefore, combining PNI with molecular markers such as KRAS, BRAF and MSI for analysis may help to more accurately stratify the risk of patients. A 2024 study involving 335 patients constructed a preoperative prediction model, revealing that tumor differentiation degree, primary tumor location, N stage as shown by CT, CEA level, and platelet/lymphocyte ratio (PLR) were independent risk factors for PNI. The model achieved an AUC of 0.772, which provides valuable assistance in assessing the possibility of PNI in patients with CRC prior to surgery (38). In recent years, the use of imaging biomarkers for predicting the PNI in colorectal cancer has become a research hotspot. An imaging biomarker model constructed based on multi-parameter MRI achieved AUC values of 0.841, 0.815, and 0.916 in the training set, internal validation set, and external validation set respectively. An external validation AUC greater than 0.9 indicates that the model has excellent generalization ability. If the radiomics model can predict PNI before surgery, patients with positive PNI can receive neoadjuvant therapy or more thorough lymph node dissection, thus avoiding the situation where the pathology is only discovered after surgery (39).

In this meta-analysis, both OS (I² = 79.7%) and DFS (I² = 67.4%) exhibited significant heterogeneity. To further explore the sources of this heterogeneity, we performed subgroup and sensitivity analyses. Subgroup analyses identified geographical region as a major source of heterogeneity. For OS, the I² was 54.6% in Asian studies versus 0% in European studies. For DFS, Asian studies showed an I² of 54.6%, while European studies reached 76.2%, with a marked difference in pooled effect sizes (Asian HR = 1.49; European HR = 3.47). Sample size also explained part of the heterogeneity: in the DFS analysis, the I² dropped from 76.2% in studies with ≤500 patients to 30.1% in those with >500 patients. Some heterogeneity probably comes from variables that weren’t consistently reported across studies, like neoadjuvant therapy use, MSI/MMR status, and different PNI scoring criteria. Variability in pathological detection methods may represent another source of heterogeneity. Most studies rely primarily on standard hematoxylin-eosin staining to assess PNI; however, this method is considerably less sensitive in identifying small nerve branches or those infiltrated by inflammatory cells (40). Previous studies have pointed out that the sole use of conventional staining may miss up to 20% to 50% of cases (41). In contrast, the implementation of S100B immunohistochemical staining significantly enhances the detection rate of tumor nerve infiltration. Patients who were overlooked using conventional staining but subsequently diagnosed with S100 staining often exhibit a markedly worse prognosis than those who are truly negative (42). Consequently, it is recommended that pathological reports routinely incorporate immunohistochemical-assisted diagnosis to minimize missed diagnoses and reduce data discrepancies between studies (42). When making clinical decisions about stage II colorectal cancer, the predictive significance of PNI is especially important. The question of whether stage II patients with negative lymph nodes should get adjuvant chemotherapy has long been debatable (43). Although there is a consensus on this issue in current international authoritative guidelines, their focuses are different. The guidelines of the National Comprehensive Cancer Network (NCCN) in the United States clearly list PNI as one of the high-risk characteristics of stage II colorectal cancer (23). It is recommended that for such patients, combination chemotherapy containing oxaliplatin or monotherapy with fluorouracil be considered (44). But the American Society of Clinical Oncology’s (ASCO) recommendations take a more cautious approach to co-decision-making (45). PNI may be helpful in guiding the selection of treatment courses for adjuvant chemotherapy in stage III colon cancer. It is worth noting that current clinical practice usually only qualitatively reports the presence or absence of PNI, but its severity may have different clinical implications. Ueno et al.’s grading method divided PNI into two categories: intramural invasion and extramural invasion. They discovered that patients with extramural PNI had a much poorer survival rate than those with intramural PNI and those without PNI (46). If this anatomical grading system can be widely applied, it will not only be able to predict prognosis more accurately, but also explain why the hazard ratio fluctuates among different studies, as different cohorts may have mixed different proportions of subtype patients (47). Therefore, in order to improve the long-term survival of these high-risk patients, future research directions should concentrate on promoting standardized testing procedures and confirming the clinical efficacy of the grading system (48). At the same time, investigate targeted therapeutic strategies for neuro-tumor signaling pathways (49).

However, this study still has certain restrictions: (1) All the included 9 studies were all of retrospective design, and their inherent risk of bias was higher than that of prospective cohort studies or randomized controlled trials. Although we evaluated the quality of the studies using the NOS score and conducted a sensitivity analysis, residual confounding factors could not be completely eliminated. (2) The combined analysis of OS and DFS both showed significant heterogeneity. Although we explored the sources of heterogeneity through subgroup analysis and the elimination method one by one, there were still some heterogeneities that could not be explained. This might be due to the inconsistencies in patient populations, the proportion of neoadjuvant therapy, and the PNI pathological interpretation criteria among different studies. However, these factors could not be systematically evaluated due to insufficient information in each study report. (3) there is a lack of quantitative analysis of the degree of PNI (such as the number of lesions and the depth of invasion). Currently, most studies only qualitatively classify it as “present/absent”. It is necessary to establish a Grading System for PNI to achieve more precise prognostic stratification. (4) The Egger test indicated that both OS and DFS were subject to significant publication bias. Although we used the trim-and-fill method for correction, the combined effect of OS after correction was no longer significant, which weakened the strength of evidence for PNI as an independent prognostic factor for OS. (5) The TNM stage of the populations included in each study varied significantly. Some studies only included patients in stages I-III, some included patients in stage IV, and some were mixed populations. Although we conducted subgroup analyses of stages, the number of studies in some subgroups was limited, making it impossible to conduct more detailed subgroup Meta-analyses. (6) We did not conduct a formal GRADE quality assessment of the evidence as it is mainly applicable to intervention studies (RCTs) and is not directly applicable to prognosis Meta-analyses of case-control and observational studies. In conclusion, the above results should be interpreted with caution in light of these limitations.

## Conclusions

PNI may be a possible predictive factor for OS and DFS in patients with colorectal cancer, although the results should be taken cautiously due to the observed heterogeneity and publication bias.

## Data Availability

The original contributions presented in the study are included in the article/**Supplementary Material**. Further inquiries can be directed to the corresponding author.
