# Supplementary material for: Prognostic and clinicopathological value of perineural invasion in colorectal cancer: a meta-analysis
Source: Front Oncol. 2026 Sep 9;16:1910736. doi: 10.3389/fonc.2026.1910736 (PMC13597262; doi:10.3389/fonc.2026.1910736)
Supplement: Supplementary file 1 [file Supplementaryfile1.zip › Supplementary Materials/Supplementary Fig.1 The Trim and fill Test of OS.pdf]

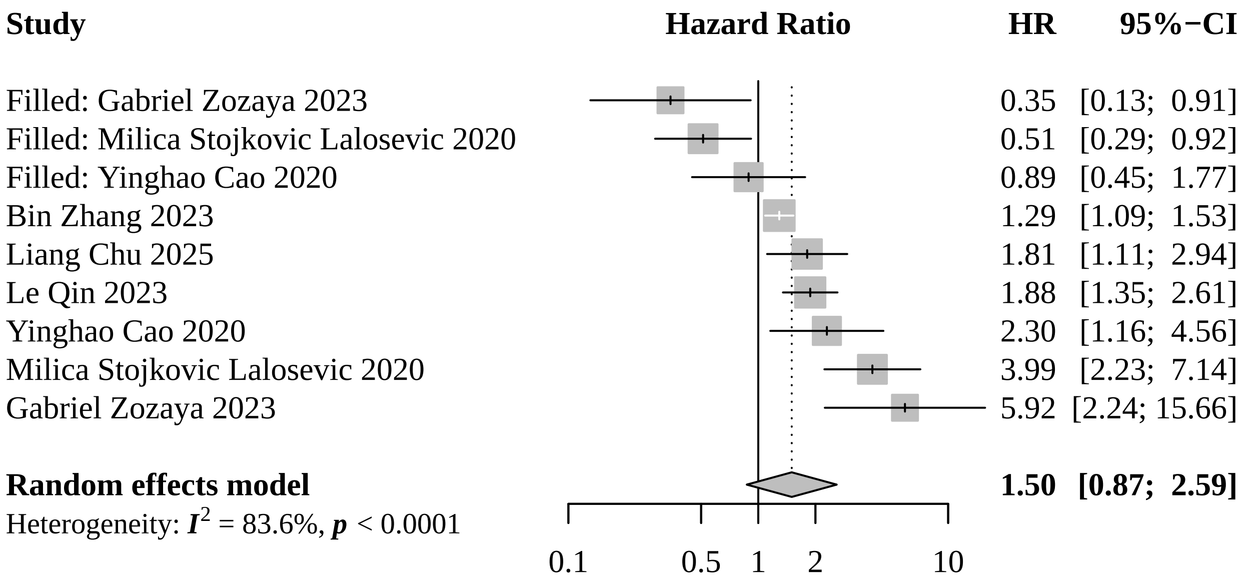
Study
Hazard Ratio
HR
95%−CI
Filled: Gabriel Zozaya 2023 0.35 [0.13; 0.91]
Filled: Milica Stojkovic Lalosevic 2020 0.51 [0.29; 0.92]
Filled: Yinghao Cao 2020 0.89 [0.45; 1.77]
Bin Zhang 2023 1.29 [1.09; 1.53]
Liang Chu 2025 1.81 [1.11; 2.94]
Le Qin 2023 1.88 [1.35; 2.61]
Yinghao Cao 2020 2.30 [1.16; 4.56]
Milica Stojkovic Lalosevic 2020 3.99 [2.23; 7.14]
Gabriel Zozaya 2023 5.92 [2.24; 15.66]
Random effects model 1.50 [0.87; 2.59]
Heterogeneity: I^2 = 83.6%, p < 0.0001
0.1
0.5
1
2
10

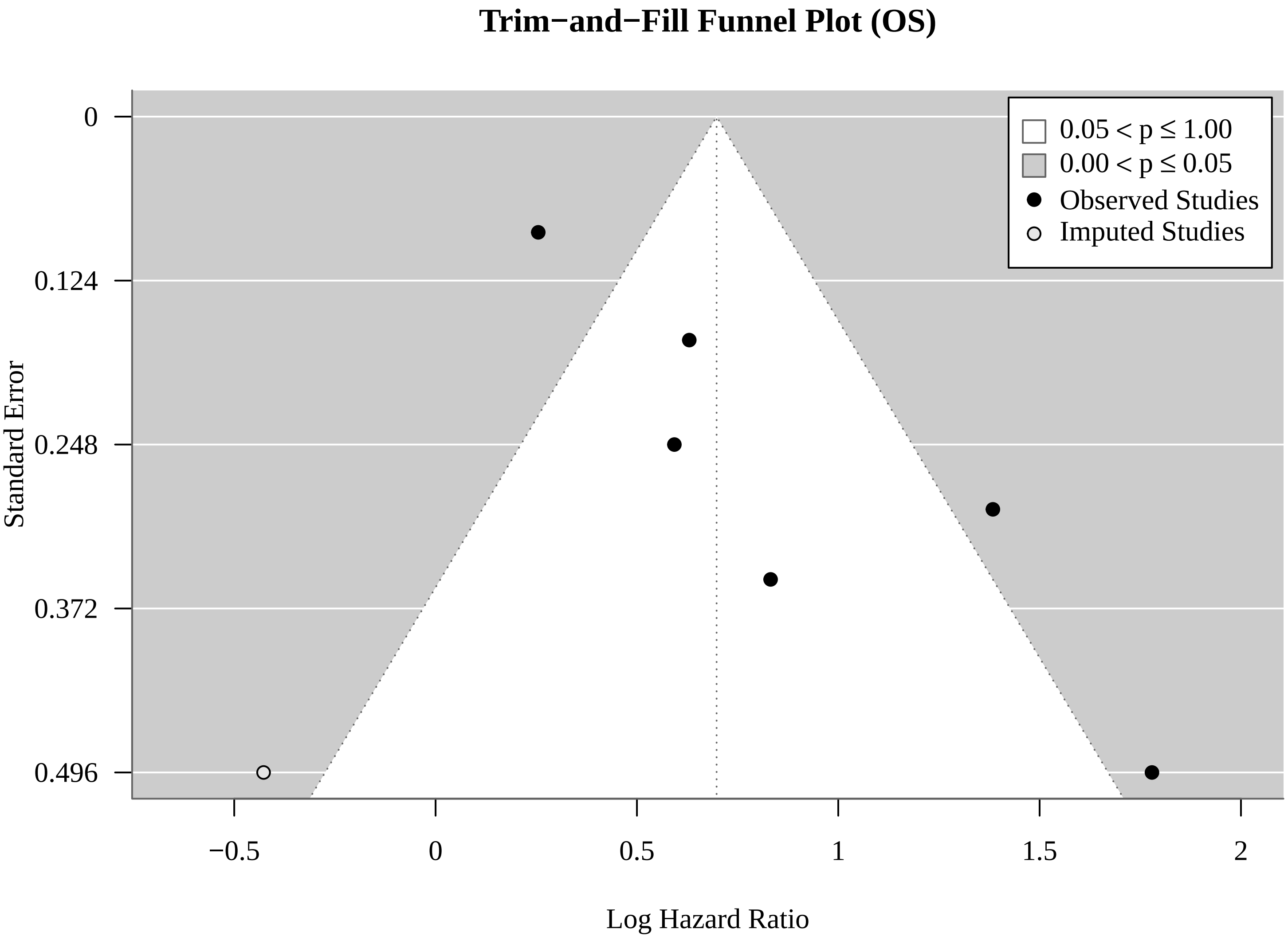
Trim−and−Fill Funnel Plot (OS)
0.05 < p ≤ 1.00
0.00 < p ≤ 0.05
Observed Studies
Imputed Studies
Standard Error
0
0.124
0.248
0.372
0.496
−0.5
0
0.5
1
1.5
2
Log Hazard Ratio
