# Supplementary material for: Prognostic and clinicopathological value of perineural invasion in colorectal cancer: a meta-analysis
Source: Front Oncol. 2026 Sep 9;16:1910736. doi: 10.3389/fonc.2026.1910736 (PMC13597262; doi:10.3389/fonc.2026.1910736)
Supplement: Supplementary file 1 [file Supplementaryfile1.zip › Supplementary Materials/Supplementary Fig.2 The Trim and fill Test of DFS.pdf]

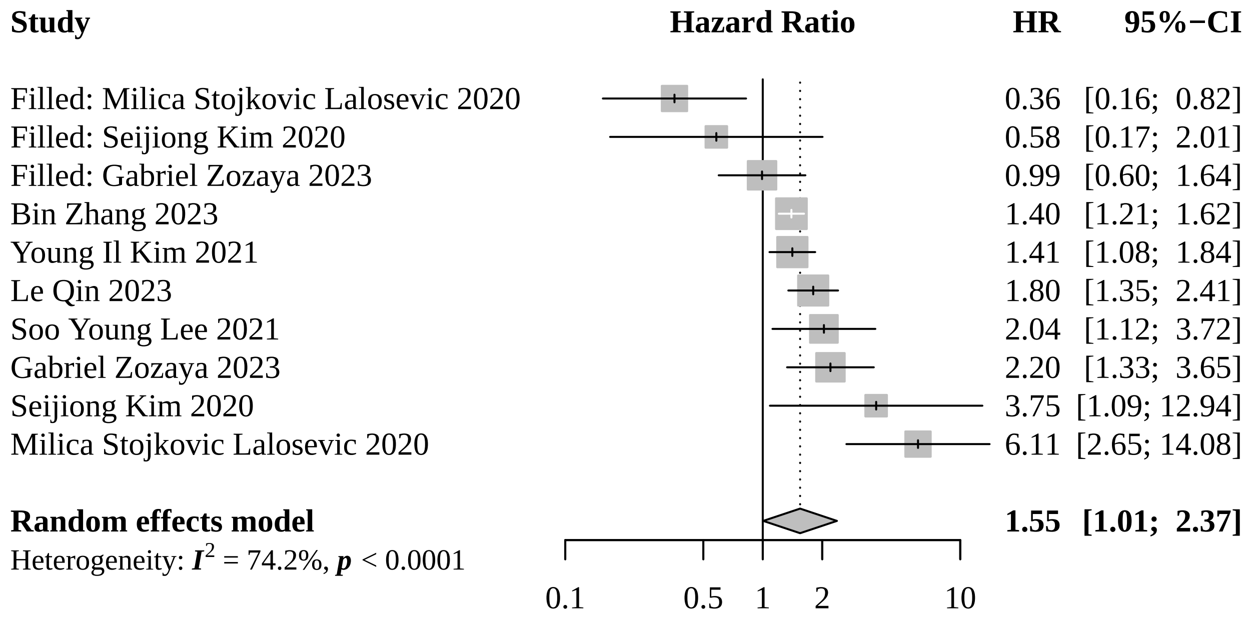
Study
Hazard Ratio
HR
95%−CI
Filled: Milica Stojkovic Lalosevic 2020 0.36 [0.16; 0.82]
Filled: Seijiong Kim 2020 0.58 [0.17; 2.01]
Filled: Gabriel Zozaya 2023 0.99 [0.60; 1.64]
Bin Zhang 2023 1.40 [1.21; 1.62]
Young Il Kim 2021 1.41 [1.08; 1.84]
Le Qin 2023 1.80 [1.35; 2.41]
Soo Young Lee 2021 2.04 [1.12; 3.72]
Gabriel Zozaya 2023 2.20 [1.33; 3.65]
Seijiong Kim 2020 3.75 [1.09; 12.94]
Milica Stojkovic Lalosevic 2020 6.11 [2.65; 14.08]
Random effects model 1.55 [1.01; 2.37]
Heterogeneity: I^2 = 74.2%, p < 0.0001
0.1
0.5
1
2
10

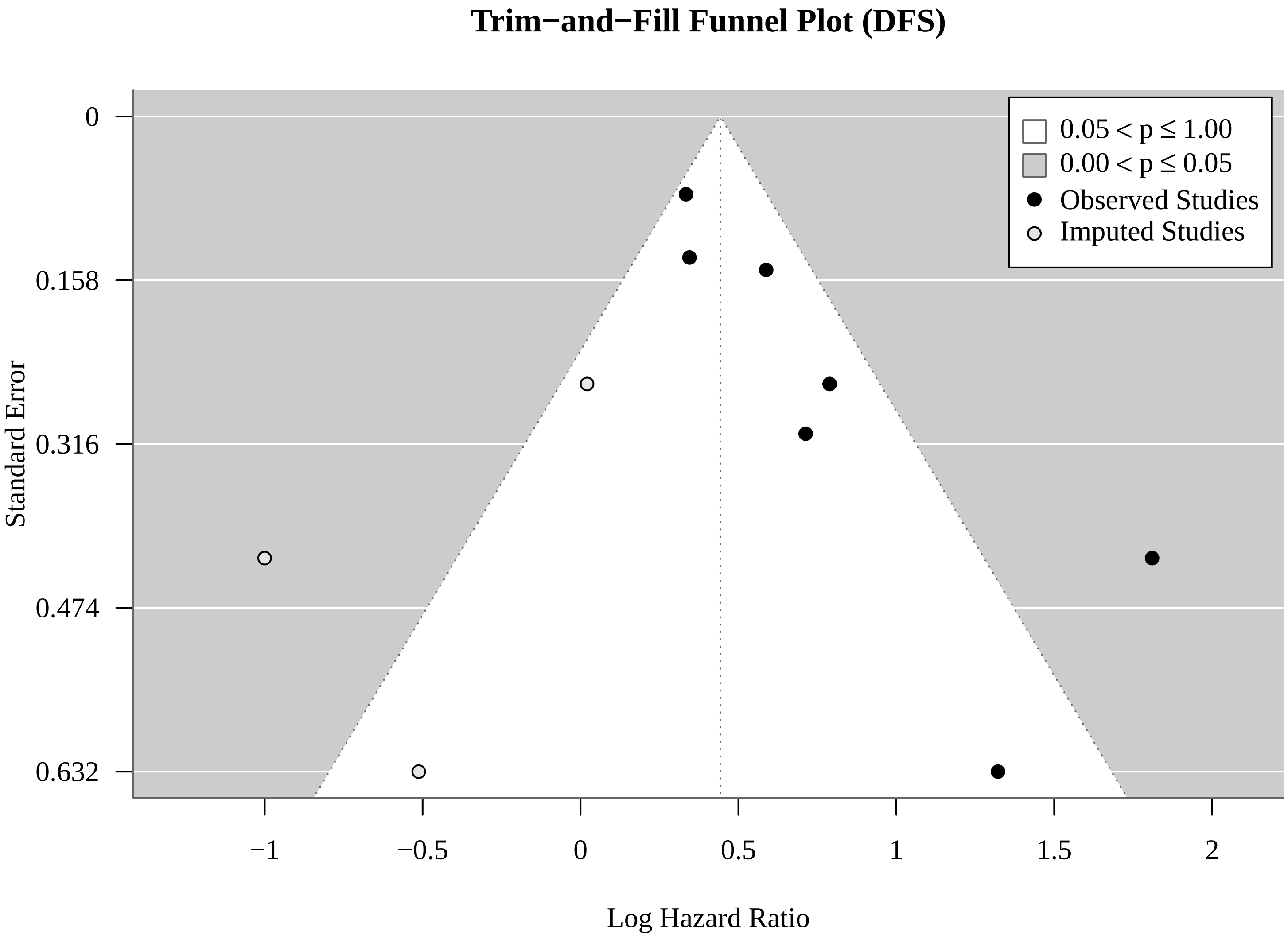
Trim−and−Fill Funnel Plot (DFS)
0.05 < p ≤ 1.00
0.00 < p ≤ 0.05
Observed Studies
Imputed Studies
Standard Error
0
0.158
0.316
0.474
0.632
−1
−0.5
0
0.5
1
1.5
2
Log Hazard Ratio
