# Supplementary material for: Prognostic and clinicopathological value of perineural invasion in colorectal cancer: a meta-analysis
Source: Front Oncol. 2026 Sep 9;16:1910736. doi: 10.3389/fonc.2026.1910736 (PMC13597262; doi:10.3389/fonc.2026.1910736)
Supplement: Supplementary file 1 [file Supplementaryfile1.zip › Supplementary Materials/Supplementary table 1-Search strategy.docx]

**Supplementary Table 1 Search strategy**

| **Database** | **Search strategy** | **Results** | |
| --- | --- | --- | --- |
| 1)PubMed  (From January 1^st^ 2020 To October 9th, 2025) | #1 | "colorectal neoplasms"[MeSH Terms] OR ("colorectal"[All Fields] AND "neoplasms"[All Fields]) OR "colorectal neoplasms"[All Fields] OR ("colorectal"[All Fields] AND "cancer"[All Fields]) OR "colorectal cancer"[All Fields]) | 98993 |
|  | #2 | (("perineural"[All Fields] OR "perineurally"[All Fields]) AND ("invasibility"[All Fields] OR "invasible"[All Fields] OR "invasion"[All Fields] OR "invasions"[All Fields] OR "invasive"[All Fields] OR "invasively"[All Fields] OR "invasiveness"[All Fields] OR "invasives"[All Fields] OR "invasivity"[All Fields])) | 3338 |
|  | #3 | ("prognosis"[MeSH Terms] OR "prognosis"[All Fields] OR "prognoses"[All Fields]) | 647001 |
|  | #4 | #1 AND #2 AND #3 | 463 |
| 2) Embase  (From January 1^st^ 2020 To October 9th, 2025) | #1 | '('colorectal cancer'/exp OR 'colorectal cancer' OR (colorectal AND ('cancer'/exp OR cancer))) | 180168 |
|  | #2 | ''perineural invasion'/exp OR 'perineural invasion') | 7697 |
|  | #3 | ('prognosis'/exp OR prognosis | 493231 |
|  | #4 | #1 AND #2 AND #3 | 186 |
| 3)Web of science  (From January 1^st^ 2020 To October 9th, 2025) | #1 | ((ALL=(colorectal cancer)) OR ALL=(colorectal neoplasms)) | 11275 |
|  | #2 | ((ALL=(perineural invasion)) OR ALL=(perineural invasion pni )) OR ALL=(perineural tumor spread ) | 3688 |
|  | #3 | (ALL=(prognosis)) | 6960 |
|  | #4 | #1 AND #2 AND #3 | 364 |
| 4) Cochrane library  (From January 1^st^ 2020 To October 9th, 2025) | #1 | (Colorectal Neoplasm) OR (Colorectal Tumor) OR (Colorectal Carcinoma) | 21618 |
|  | #2 | "perineural invasion" | 200 |
|  | #3 | "prognosis" | 49513 |
|  | #4 | #1 AND #2 AND #3 | 7 |
