# Supplementary material for: Prognostic and clinicopathological value of perineural invasion in colorectal cancer: a meta-analysis
Source: Front Oncol. 2026 Sep 9;16:1910736. doi: 10.3389/fonc.2026.1910736 (PMC13597262; doi:10.3389/fonc.2026.1910736)
Supplement: Supplementary file 1 [file Supplementaryfile1.zip › Supplementary Materials/Supplementary table2 Quality assessments of included studies based on Newcastle–Ottawa scale.docx]

Supplementary table 2; Quality assessments of included studies based on Newcastle–Ottawa scale

| Author；  Publication Year | Selection | | | | Comparability | Outcome | | | Total  score |
| --- | --- | --- | --- | --- | --- | --- | --- | --- | --- |
|  | Representativeness  of the exposed  cohort | Selection  Of  The nonexposed  cohort | Ascertainment of  exposure | Demonstration that outcome of interest was not present at start of  study | Comparability  of cohorts on the basis of the design or analysis | Assessment  of outcome | Was follow-up long enough for  outcomes to occur | Adequacy of follow up of cohorts |  |
| Liang Chu 2025 | 1 | 1 | 1 | 0 | 2 | 1 | 0 | 0 | 6 |
| Yinghao Cao 2025 | 1 | 1 | 1 | 0 | 2 | 0 | 1 | 1 | 7 |
| Milica Stojkovic Lalosevic 2020 | 1 | 1 | 1 | 0 | 2 | 1 | 1 | 1 | 8 |
| Soo Young Lee 2021 | 1 | 1 | 1 | 0 | 2 | 1 | 1 | 1 | 8 |
| Le Qin 2023 | 1 | 1 | 1 | 0 | 2 | 0 | 1 | 0 | 6 |
| Seijong Kim 2020 | 1 | 1 | 1 | 0 | 2 | 1 | 1 | 0 | 7 |
| Gabriel Zozaya 2023 | 1 | 1 | 1 | 0 | 2 | 1 | 0 | 0 | 6 |
| Bin Zhang 2023 | 1 | 1 | 1 | 0 | 2 | 1 | 1 | 0 | 7 |
| Young Il Kim 2021 | 1 | 1 | 1 | 0 | 2 | 1 | 1 | 1 | 8 |
